# Integrated Hypertension-HIV Care in Botswana: Mixed-Methods Evaluation of Determinants of Success in an Implementation Trial

**DOI:** 10.1007/s43477-025-00178-2

**Published:** 2025-07-17

**Authors:** Amelia Van Pelt, Moagedi Mawi, Keonayang Kgotlaetsile, Mareko Ramotsababa, Nabila Youssouf, Ponego Ponatshego, Edwin Mogaetsho, Tendani Gaolathe, Thato Moshomo, Onkabetse Julia Mofefe-Baikai, Laura M. Bogart, Evelyn Dintwe, Shabbar Jaffar, Kago Kebotsamang, Lisa R. Hirschhorn, Mosepele Mosepele

**Affiliations:** 1 Northwestern University, Evanston, USA; 2 University of Botswana, Gaborone, Botswana; 3 Ministry of Health, Gaborone, Botswana; 4 London School of Hygiene & Tropical Medicine, London, UK; 5 Botswana Harvard Health Partnership, Gaborone, Botswana; 6 RAND Corporation, Santa Monica, USA; 7 Charles R. Drew University of Medicine and Science, Los Angeles, USA; 8 University College London, London, UK

**Keywords:** Patient centered care, Hypertension management, HIV care, Treatment partners, Integration

## Abstract

Due to the success of HIV treatment, people living with HIV (PWH) have aged to develop chronic conditions. InterCARE is evaluating the integration of hypertension treatment into HIV care in Botswana. This involves adapting the Electronic Health Records [EHR], health care workers training, and the use of treatment partners. A pilot hybrid type 2 effectiveness implementation trial in two public HIV clinics found high effectiveness, adoption, and fidelity overall but low adoption of EHR. This mixed-methods research aimed to explain mean variation in these outcomes to inform future scale-up. Guided by the Consolidated Framework for Implementation Research, we recruited community members, healthcare workers, patients, and treatment partners to complete surveys (*n* = 100), and semi-structured interviews in-person (*n* = 10), 12 months after InterCARE implementation. Descriptive statistics and Fischer’s exact tests were calculated for surveys, and a deductive analysis was completed for interviews (Cohen’s Kappa = 0.86). Qualitative analysis found high acceptability of InterCARE compared to standard of care for HIV and hypertension. Integration improved patient experiences by increasing efficiency of care (e.g., reduced visit frequency, less time spent accessing care, reduced out-of-pocket costs). Integration of care and use of treatment partners increased medication adherence. Barriers included insufficient resources, HIV-related stigma (both internalized and perceived), high healthcare workers workload, and need for HIV providers’ training in hypertension. To improve implementation, suggestions included increasing client awareness and understanding of services through education, using various dissemination strategies (e.g., social media, community meetings, handouts), and enhancing provider buy-in. Overall, results showed positive perceptions of the strategies and overall integration. This research provides valuable insight into factors that affect outcomes from bundled implementation strategies supporting integration among chronic diseases and HIV in resource-limited settings.

Due to advances in antiretroviral treatment (ART), people living with HIV (PWH) now have a near normal lifespan. In Botswana, a country with the third highest HIV prevalence (Agency, 2022), success in the scale-up of HIV care has surpassed the World Health Organization (WHO) 95–95-95 goals (Mine et al., 2024). This success has incorporated strategies including the use of treatment partners (also known as “adherence buddies”; informal social network member selected by a patient) (Bogart et al., 2018; Ramiah & Reich, 2005), standardized national training (Bussmann et al., 2008; Ledikwe et al., 2013), and development of an HIV-related Electronic Health Records (EHR) (Galani et al., 2021). As PWH survive longer, aging among the population has led to the development of co-occurring chronic conditions (Xu et al., 2017). For example, approximately one-third of PWH in Botswana are estimated to have hypertension (Mosepele et al., 2023), which increases the underlying risk for the development of cardiovascular disease. Although evidence-based practices to manage hypertension exist in Botswana, studies report low awareness (only 46% of individuals with hypertension are aware of their diagnosis), low access to treatment (42% of those diagnosed are on hypertension medication), and poor blood pressure control (44% of people with controlled blood pressure) (Mosepele et al., 2023). Efforts to improve adoption of integrated care across the hypertension care cascade are needed in this setting.

The InterCARE (Integrating Hypertension and Cardiovascular Care into Existing HIV Services Package) intervention was developed to increase the uptake of evidence-based practices for hypertension control among PWH in Botswana (Youssouf et al., 2024). HIV treatment in Botswana in the public sector is provided through Infectious Disease Care Clinics (IDCC’s) (Wester et al., 2005). Given that most PWH in Botswana receive care at the IDCCs, these sites offer an optimal location for the integration of hypertension services. Leveraging the success of HIV care in Botswana, InterCARE integrates hypertension treatment into IDCCs through three evidenced-based strategies: (1) adaptation of the existing HIV EHR to capture hypertension risk and treatment, (2) facilitation of provider training on hypertension diagnosis and management, and (3) use of treatment partners to support adherence of hypertension care. Utilizing generalizable nomenclature from implementation science, these strategies align with the following strategies in the Expert Recommendations for Implementing Change (ERIC) compilation (Powell et al., 2015), a classification of methods that can be used to enhance implementation of a clinical intervention: (1) “change record system,” (2) “conduct educational meetings,” and (3) “involve patients/consumers and family members” and “intervene with patients/consumers to enhance uptake and adherence, respectively.”

A pilot type 2 hybrid effectiveness-implementation trial was conducted to examine implementation of InterCARE in two IDCCs. The trial found high effectiveness, as blood pressure control significantly improved (Moshomo et al., 2024). In addition, the strategies achieved high adoption for providers doing counselling related to medication, diet and exercise (Moshomo et al., 2024). However, adoption for EHR components was poor, including documentation of blood pressure medication prescription or cardiovascular risk factor evaluation in the EHR.

To facilitate the scale-up of InterCARE, an understanding of implementation determinants to explain these outcomes is needed (Lane-Fall et al., 2019). The Consolidated Framework for Implementation Research (CFIR) provides a framework for the systematic evaluation of factors that may affect implementation across multiple ecological levels (Damschroder et al., 2022). CFIR consists of five domains: innovation (e.g., components of InterCARE), inner setting (e.g., clinic leadership structure), outer setting (e.g., policy context, community stigma), individuals (e.g., self-efficacy), and implementation process (e.g., strategies). This research conducted contextual inquiry to provide insight into the success of InterCARE strategies and where adaptation was needed before scaling up InterCARE in a planned large-scale hybrid effectiveness implementation trial.

## Methods

The Consolidated Criteria for Reporting Qualitative Research 1.0 (Tong et al., 2007) and the Standards for Reporting Implementation Studies (StaRI) Statement (Pinnock et al., 2017) guided the reporting of this research (Supplemental File 1 and Supplemental File 2). All procedures were approved by the Institutional Review Boards at the University of Botswana and the Health Research and Development Committee within the Botswana Ministry of Health (reference: HPRD 6/14/1). This manuscript presents findings from the 12-month follow-up assessment of a pilot study that was previously assessed at baseline (Gala et al., 2024). Additionally, we followed the same protocol framework as the baseline and used similar surveys and a small number of interviews to assess changes and implementation experience over time. This pilot was conducted as part of the larger trial described by Youssouf et al. (2024) and followed the same study protocol.

### Study Setting

To optimize understanding of contextual factors, IDCCs were stratified by size (e.g., small rural serving less than 500 patients versus large peri-urban serving more than 1,500 patients) and urbanicity. Two IDCCs, one from each of the stratification categories, were randomly selected for participation in the study: small clinic serving < 500 patients in the rural south of Botswana) and large clinic serving more than > 1,500 patients in the peri-urban Northeast of Botswana). Small clinic primarily employs nurses, whereas a large clinic employs general nurses, physicians (medical officers), and nurse practitioners (including HIV nurse prescribers).

### Quantitative Data

#### Survey Participants

To capture the diversity of perspectives, participants were recruited from four constituent groups: community members (e.g., local leaders, council members, and leaders in the commercial sector), healthcare workers, patients, and HIV treatment partners of the same patients (See Supplemental File 3 for an overview of participant groups, recruitment methods, and relationship to baseline and 12-month follow-up timepoints). Participants were required to be 20–75 years old. Patients were defined as individuals with a dual diagnosis of both HIV and hypertension who were enrolled for care at one of the study sites. Participants were recruited from the same pool of patients enrolled in the InterCARE pilot who were enrolled in the baseline surveys, so some participated in both baseline and endline, but some were only in the endline. All eligible patients, treatment partners, and healthcare workers were recruited in-person at each site until the target sample was achieved. Community members were eligible if they were aged 20 or older, resided in one of the study areas and were not InterCARE participants or treatment partners. These participants were purposively recruited based on their role in the community (e.g. local leaders, council members, or individuals in the commercial sector). Participants were identified and invited to participate based on their community roles until the target sample was reached at each study site. Sampling aimed to recruit more community members than other constituent groups, as their perspective was particularly important for adaptations to InterCARE in future scale-up. Participants who completed the endline surveys differed from participants who completed the baseline surveys.

#### Survey Procedures

The CFIR guided the development of the survey instrument (Damschroder et al., 2022). A subset of CFIR constructs within the “innovation” domain were selected based on perceived factors relevant to implementation success: innovation design, compatibility, relative advantage, adaptability, and complexity. In addition, two survey items corresponded to the “inner setting” domain construct of “structural characteristics.” The final instruments included 22 items (See Supplemental File 4 for an example survey). The endline surveys included the same items as the baseline surveys, but the wording was changed to reflect the temporality of post-implementation. All instruments were available in both English and Setswana, the local language in Botswana. The survey instruments were pre-pilot tested in two clinics that had characteristics closely matching those of the pilot study sites and subsequently used pre-implementation (Gala et al., 2024). The survey was administered immediately after the month 12 visit of the InterCARE pilot (post-implementation).

Written informed consent and demographic information were obtained from all participants. Surveys were administered in-person in a private room in the study clinics (or private community-based office used by community member) by a member of the research team. Participants had the option of completing the survey in English or Setswana. On average the interviews were 30–45 min long.

### Qualitative Data

#### Interview Participants

Similar to the survey participants, qualitative interviewees were purposively selected from the four constituent groups: community members, healthcare workers, patients, treatment partners. The aim was to recruit four participants from the community and two each from the other three groups based on resource and time constraints. For healthcare workers, patients, treatment partners recruitment targeted individuals who had been involved with InterCARE for at least 12 months to ensure enough exposure to the intervention. While endline participants were not the same individuals as the baseline participants, they were drawn from the same clinical population. Written informed consent and demographic information were obtained from all participants. The intentions of the investigators were shared during this recruitment process. Participant refusal and dropout did not occur.

#### Interview Procedures

The CFIR guided the development of semi-structured interview guides for each constituent group (See Supplemental File 5 for an example guide). The interview guides were pretested among 10 participants, with 2 participants selected from each group. No changes were made to the interview guide based on pre-testing.

All interviews were conducted in-person in a private room in the study clinic by research assistants trained in qualitative research with relevant expertise: KBM (Motswana, female, BSc in Health Education and Promotion), KM (Motswana, female, Certificate in social work), TS (Motswana, female, Bachelor of Public Health), and BN (Motswana, male, Diploma in Health Education and Promotion). The interviewers did not have prior relationships with the participants. Participants had the option of being interviewed in English or Setswana. All interviews were audio-recorded, transcribed, and translated into English, if needed, by a member of the research team. The transcripts were uploaded into Dedoose (v9.0.107; Dedoose, 2023) for data management and analysis.

### Data Analysis

A convergent mixed methods analysis approach was used (Palinkas et al., 2011). First, the survey and interview data were analyzed separately.

#### Quantitative Data

To facilitate greater understanding of perspectives, the five-point Likert scale for the survey items was collapsed into a dichotomous outcome (DiStefano et al., 2021): Agree (“strongly agree” and “agree”) versus Do not Agree (“neutral,” “disagree,” and “strongly disagree”). Items were reverse coded as needed so lower numbers indicate higher agreement. Descriptive statistics were calculated on participant demographic data for both the surveys and interviews, as well as survey data on agreement stratified by constituent group. To compare the baseline data to the endline data, Fischer’s exact tests were conducted. All analyses were completed in R (v4.3.2; R Core Team, 2023).

#### Qualitative Data

A deductive analysis was conducted (J). The codebook comprised constructs from CFIR, barriers, and facilitators that were identified a priori. An initial coding scheme was applied to two transcripts by two independent coders (AVP and KK).

Given the small sample size, double coding of 20% of the transcripts included the two initial transcripts to ensure consistency and refine the coding framework before proceeding with the full analysis. Disagreements were resolved through team discussions and after resolving them the pair achieved reliability (Cohen’s Kappa = 0.86) (Saldana, 2009). The final coding scheme was applied to all transcripts by one investigator. However, three members of the research team (AVP, KK, LB) reviewed all coded transcripts to confirm the accuracy of themes. All constituent groups were analyzed together.

## Results

### Participant Characteristics

#### Surveys

One hundred participants completed the endline surveys. As planned, these included community members (41.0%), patients (20.0%), treatment partners (20.0%), and healthcare workers (19.0%); distribution by clinic was approximately equal (Table 1).

#### Interviews

Fifty-eight (58) participants completed interviews from the fourteen (14) study sites. The interview duration was 30–45 min. As planned, four were community members and two from each of the other three groups (healthcare workers, patients, treatment partners, community members) (Table 2). Most participants were female (80%). Median age was 42 years (IQR: 25–59 years).

### Themes by CFIR Domain

Table 3 presents survey data, and Table 4 highlights the main qualitative themes organized by CFIR domain. (See Supplementary Material 6 for a pre-post comparison.)

#### Innovation Domain

##### Quantitative Data.

Perspectives on the design of InterCare varied by constituent group (Table 3). Treatment partners and healthcare workers indicated high agreement in the value of the intervention, with (*n* = 20, 100.0%) and (*n* = 20, 94.7%) respectively agreeing that InterCARE would be successful in improving treatment of people with HIV and hypertension. More than half of healthcare workers reported the importance of the treatment partner (*n* = 18, 94.7%), EHR (*n* = 17, 89.5%), and provider training (*n* = 16, 84.2%) components of InterCare. Almost all (*n* = 40, 97.6%) of community members also reported high opinions of InterCARE’s design, in terms of success in improving patient knowledge about managing high blood pressure. Additionally, (*n* = 35, 85.4%) agreed that “combining high blood pressure and HIV care in the same clinic visit will improve a patient’s blood pressure.”

Opinions on InterCARE’s compatibility differed by respondent type. Three quarters (*n* = 15, 78.9%) of healthcare workers reported high compatibility, defined as being compatible and consistent with the needs of people with HIV and hypertension in [their] clinic. Similarly, almost all community members and treatment partners shared high acceptability and compatibility of treatment partners, reflecting the high adoption seen. For example, participants agreed that a treatment partner for high blood pressure will help patients (1) remember to go to appointments (community members: *n* = 40,97.6%; treatment partners: *n* = 20, 100.0%); (2) remember to take their medications (community members: *n* = 40, 97.6%; treatment partners: *n* = 19, 95.0%); and (3) to make changes to their diet to reduce blood pressure (community members: *n* = 40, 97.6%; treatment partners, *n* = 20, 100.0%); and (4) to increase their physical activity to reduce blood pressure (community members: *n* = 40, 97.6%; treatment partners, *n* = 19, 95.0%).

Healthcare workers’ perspectives on InterCARE’s relative advantage were also strong, with (*n* = 18, 94.7%) agreeing that InterCARE would be more effective than interventions currently used to manage hypertension in PWH in their clinic.

In contrast, and despite the high uptake reported, more participants believed that the complexity of InterCARE would pose some challenges. For example, (*n* = 11, 57.9%) of healthcare workers agreed that “InterCARE is too complex to put into place in [their] clinic,” and (*n* = 29, 70.7%) of community members agreed that combining high blood pressure and HIV care into the same clinic visit would be too complicated. These increases suggest that while uptake of the intervention was high, real-world implementation revealed additional operational and structural challenges not fully anticipated at baseline.

##### Qualitative data.

Interview themes and quotes are shown by CFIR domain in Table 4. Qualitatively, Participants discussed that InterCARE improved access to health services and patient experiences. For example, a healthcare provider highlighted InterCARE’s alignment with the clinic’s value of patient-centeredness. Integration was also perceived to increase the efficiency of care through the reduction in the number of clinic visits and time to see providers. As a result, time and costs associated with traveling to appointments decreased, as reflected in high patient retention. The InterCARE model also made it easier for patients to take medications, both using treatment partners and through the ability to pick up all their medications in one location.

#### Outer Setting

Participants were aware of a need for improving hypertension care based on the high prevalence of hypertension in Botswana, and said that, in contrast to HIV, limited stigma for high blood pressure existed in the community. Participants explained that typical services for hypertension and HIV are usually provided in different settings (e.g., separate clinics), which resulted in barriers such as far distances to travel to attend clinic visits.

### Inner Setting

#### Quantitative data.

Overall, (*n* = 7, 36.8%) healthcare workers felt that InterCARE required too many staff or other resources. A similar percentage of healthcare workers (*n* = 8, 42.1%) felt that there were insufficient resources to expand care for any additional PWH with hypertension.

#### Qualitative data.

While healthcare providers were familiar with integration both through InterCARE and other conditions and had positive responses regarding the strategies, there were concerns related to availability of resources. For example, in some settings (clinics), necessary equipment (e.g., blood pressure machines and weighing scales) and medications for hypertension were not available. Power outages also created barriers to adoption to the EHR. Further, space constraints were a concern if the integration of services resulted in an additional influx of patients. Participants also expressed that the integration of services could result in a greater workload for each individual provider, and therefore, create a longer queue of patients waiting to see a specific individual.

##### Individuals

Further supporting the innovation domain, multiple constituent groups (providers, patients, treatment partners) expressed high acceptability of InterCARE. Participants highlighted that InterCARE provided value through benefitting patients. Further, participants expressed confidence in the capacity and abilities of the team to effectively engage and benefit from the study. One challenge related to the training of providers, as some participants explained that healthcare workers working in HIV may not have sufficient training in hypertension required for facilitating dual care.

##### Implementation Process

Both treatment partners and healthcare providers stressed the importance of more education and engagement of multiple constituent groups. For the community, participants explained the importance of education to increase awareness and, consequently, acceptability of InterCARE. They emphasized education’s influence on contributing to the improvement of health in village settings and increasing receptiveness to health programs. To facilitate the increase in awareness, participants suggested disseminating information via kgotla meetings (i.e., traditional community meetings), social media, radio, WhatsApp, and clinics. Participants also emphasized the importance of receiving buy-in from key individuals such as the District Health Management Teams to champion the intervention. To achieve critical buy-in from matrons (i.e., leaders of the clinics), participants recommended communicating the details of InterCARE to facilitate understanding and value of the intervention. Thinking about sustainability and scale, Participants also suggested that matrons should help to lead and monitor implementation of InterCARE, such as the procurement of funds and resources, as well as scale-up to other regions.

##### Adaptation of Strategies

Additional barriers included lack of treatment guideline availability, staff turnover with need for repeat training, and need to strengthen clinic leadership in performance improvement. Several strategies were adapted or introduced in response prior to the start of the full scale InterCARE study. For example, the main gap was in EHR uptake, and in addition to power issues, the absence of new hypertension medications as options in the EHR also limited adoption. In response, the EHR was updated, and the team worked to strengthen electrical power for new study sites. In addition, to increase clinic oversight and leadership, monthly site audit reports will be generated from study data and the EHR to facilitate discussions with sites on performances and challenges in EHR use and other areas. To address the scarcity of treatment guidelines, all facilities will be provided with a blood pressure treatment guidelines algorithm (e.g., posters as reminders and decision support). To improve education among participants and increase adherence to medications and clinic visits, collaboration with other organizations working in the clinics to facilitate health education was planned.

## Discussion

Using mixed methods, this research identified multiple factors that helped explain the effectiveness and implementation outcomes of the InterCARE Project integrating hypertension into HIV care. Facilitators included the compatibility of integration, use of treatment partners, and the healthcare workers training. Despite the overall effectiveness and high retention, some barriers were identified related to concern about resources and increased workload and waiting times. Consistent with work across Africa, integrating hypertension care into HIV service delivery platform is one of the most common forms of integration of HIV/NCD integration in Africa (van der Mannen et al., 2024), which might explain the overall positive feedback from InterCARE pilot.

These findings corroborate those reported in a similar setting in southern Malawi. In a mixed methods study that assessed Hypertension, Diabetes Mellitus and dyslipidemia integration into five HIV clinics in Malawi, use of existing HIV infrastructure, healthcare workers training, patient information about integration, and time efficiency were seen as facilitators of CVD care into HIV service delivery platform (Katundu et al., 2024). Like the InterCARE study, lack of knowledge of CVD by healthcare workers and increased workload were seen as potential barriers. In another study of 14 adults with HIV and hypertension in Somalia, they also revealed that “support from family”, which is similar to the modified motivational interviewing-trained treatment partner in InterCARE, enhanced social support from the patient social network (Badacho & Mahomed, 2024). Consistent with high acceptability of InterCARE, a study from Somalia identified “lack of integration” as a significant barrier to services for PWH/hypertension- a barrier that the InterCARE strategies aims to remove. Aligned with the InterCARE results, a recent narrative review of 14 publications from Africa on integration of HIV/NCDs reported that training of both healthcare workers and patients were critical (van der Mannen et al., 2024).

Qualitative and quantitative findings provide additional insight into possible explanations for the implementation outcomes, such as poor adoption of the EHR components in the pilot trial. The contextual inquiry highlighted perceptions of high complexity of InterCARE and healthcare workers insufficient training in hypertension, both of which may have resulted in lower willingness or self-efficacy to use these components of the EHR. This observation is similar to the low (11%) adoption of technologies for integrated non-communicable disease care in primary health care setting in Africa (Tesema et al., 2020). These perceptions were present at baseline (Gala et al., 2024), where one-quarter of healthcare providers and nearly one-third of community members believed the intervention would be too complex to implement. Concerns were even higher regarding the use of treatment partners. Notably, these concerns persisted at endline, with over half of healthcare workers and nearly three-quarters of community members reporting that combining HIV and hypertension care was too complicated. In our context, participants perceptions of training were shaped by their experience with routine care, which did not include content on hypertension and cardiovascular care management (Moshomo et al., 2024). These experiences occurred prior to the introduction of the InterCARE intervention. Similarly, studies across sub-Saharan Africa suggest that while integrating hypertension into HIV care can enhance efficiency and patient-centeredness when supported by structured models and capacity building (e.g. integrated visits in Uganda (Muddu et al., 2022) or quality improvement collaborative in Namibia (Basenero et al., 2022), these benefits are undermined unless accompanied by training, reliable supplies, and clear workflows.

At the endline, both qualitative and quantitative data from our InterCARE pilot trial found sustained impact for the treatment partner component compared to baseline perceptions which highlighted familiarity and structural fit. Participants reported high fidelity to the treatment partner strategy, perceived value, and confidence in their effectiveness, and high overall acceptability of InterCARE, all of which could have contributed to willingness to implement. The observed enthusiasm to adopt the strategy may also be shaped by prior experiences with Mopatis, a nationally recognized treatment partner initiative. Studies such as (Bogart 2024; Nyoni 2020; Nakamanya 2019; Bogart 2018) have documented the effectiveness of using treatment partners in Botswana’s HIV care.”

Further, our InterCARE pilot trial had high reach overall (Moshomo et al., 2024), which may have resulted from the high acceptability, positive perceptions of integration of care, and minimal barriers to implementation. Overall, participants were satisfied with how InterCARE was implemented including the training but recommended some adaptations to implementation strategies. In addition, the pilot trial achieved high adoption for the counselling-related components, specifically related to diet and exercise (13). Our results suggest that the training intervention strategy may have contributed to higher counselling by healthcare workers. For instance, in a prior cross-sectional study of 3,981 adults across Botswana, of whom 30% were PWH, counselling was 42% for salt (diet) and 46% for physical activity/exercise. Therefore, our training implementation strategy almost doubled rates of counselling for lifestyle associated risk factors for cardiovascular disease (Molefe-Baikai et al., 2024). The contextual inquiry highlighted a need for promotion of healthy eating and exercise, which may reflect the interest in these topics and, consequently, pursuit of counselling. HIV providers described lack of prior or ongoing training in NCD care in Botswana’s Primary Health Care setting which contributed to lower rate of counselling for medication self-efficacy (Tesema et al., 2020). The findings provided insightful input for the adaption of strategies before initiation of a scaled-up hybrid type 2 study.

### Limitations

This research had several limitations. First, the sample size for the semi-structured interviews was too small for comparison of themes across constituent groups (Hennink & Kaiser, 2022), although thematic saturation was achieved. Furthermore, recruitment was limited to those with a known diagnosis and were actively engaged in care, the findings may not be generalizable to individuals who are undiagnosed or not currently engaged in care. Second, collapsing the survey response options from a five-point Likert scale to a dichotomous outcome minimizes the nuance in the data. Third, the surveys only contained items corresponding to the CFIR “innovation” domain, with two items for “inner setting.” This quantitative information provided valuable information, but the limited data prevented a mixed-methods analysis for all domains. Nevertheless, this research provided valuable insight into factors that affect bundled implementation strategies among people with HIV and hypertension in resource-limited settings, as well as scalability into new settings and clinical conditions.

## Conclusion

The mixed methods helped explain the outcomes in the pilot implementation of InterCARE, as well as areas where adaptation of strategies was needed. In response, the team has made adaptations before scaling up to the large-scale hybrid effectiveness-implementation trial. Thus, this research provided valuable insight into factors that affect bundled implementation strategies among PWH and diagnosis of hypertension in resource-limited settings, as well as scalability into new settings and clinical conditions.

## Supplementary Material

Supplemental material

**Supplementary Information** The online version contains supplementary material available at https://doi.org/10.1007/s43477-025-00178-2.

## Figures and Tables

**Table 1:** Post intercare pilot survey participant characteristics

Variable	Patients			Healthcare workers			Treatment partners			Community		
				
	N (%)			N (%)			N (%)			N (%)		
				
	Pre	Post	*P*-value	Pre	Post	*P*-value	Pre	Post	*P*-value	Pre	Post	*P*-value

**Total**	**20**	**20**		**20**	**19**		**20**	**20**		**40**	**41**	
**Gender**												
Male	10(50.0)	4(20.0)	0.096	9(45.0)	7(36.8)	0.75	1(5.0)	1(5.0)	>0.99	17(42.5)	17(41.5)	>0.99
Female	10(50.0)	16(80.0)		11(55.0)	12(63.2)		19(95.0)	19(95.5)		23(57.5)	24(58.5)	
**Age (years)**												
21–30	3(15.0)	4(20.0)	0.89	2(10.0)	4(21.1)	0.88	4(20.0)	1(5.0)	0.19	4(10.0)	8(19.5)	0.56
31–40	4(20.0)	2(10.0)		7(35.0)	6(31.6)		4(20.0)	8(40.0)		17(42.5)	17(41.5)	
41–50	5(25.0)	5(25.0)		8(40.0)	7(36.8)		7(35.0)	3(15.0)		12(30.0)	8(19.5)	
>50	8(40.0)	9(45.0)		3(15.0)	2(10.5)		5(25.0)	8(40.0)		7(17.5)	8(19.5)	
**Education level**												
Less than primary	5(25.0)	3(15.0)	0.42	0(0)	0(0)	>0.99	0(0)	1(5.0)	0.65	0(0)	3(7.3)	**0.05**
Primary school	3(15.0)	6(30.0)		0(0)	0(0)		5(25.0)	5(25.0)		4(10.0)	4(9.7)	
Junior secondary	7(35.0)	7(35.0)		0(0)	0(0)		10(50.0)	6(30.0)		17(42.5)	7(17.1)	
Senior secondary	4(20.0)	1(5.0)		0(0)	0(0)		3(15.0)	4(20.0)		9(22.5)	16(39.0)	
Higher than Senior Secondary	1(5.0)	3(15.0)		20(100)	19(100)		2(10.0)	4(20.0)		10(25.0)	11(26.8)	
**Employment Status**												
Employed	5(25.0)	4(20.0)	>0.99	20(100)	19(100)	>0.99	10(50.0)	7(35.0)	0.52	35(87.5)	38(92.7)	0.48
Unemployed	15(75.0)	16(80.0)		0(0)	0(0)		10(50.0)	13(65.0)		5(12.5)	3(7.3)	
**Monthly Income in Pula** *												
<1,000	9(45)	17(85.0)	**0.03**	-			11(55.0)	12(60.0)	0.65	9(22.5)	11(26.8)	0.09
1,000–4,999	6(30)	2(10.0)		-			7(35.0)	7(35.0)		11(27.5)	18(43.9)	
≥5,000	1(5)	1(5.0)		-			2(10.0)	0(0)		10(25.0)	10(24.4)	
Prefer not to answer	1(5)	0(0)		-			0(0)	0(0)		2(5.0)	1(2.4)	
Don’t know	3(15)	0(0)		-			0(0)	1(5.0)		8(20.0)	1(2.4)	
**Clinic Location**												
S mall clinic (rural)	10(50.0)	10(50.0)	>0.99	10(50.0)	9(47.4)	>0.99	10(50.0)	10(50.0)	>0.99	21(52.5)	20(48.8)	>0.99
Large clinic (peri-Urban)	10(50.0)	10(50.0)		10(50.0)	10(52.6)		10(50.0)	10(50.0)		19(47.5)	21(51.2)	

Note.

* 1 Pula to 0.07 USD, healthcare workers: Health care workers. No data was collected on income for Health care workers

**Table 2:** Interview participant characteristics

Variable	N (%)

**Total**	10
**Median Age (IQR)** ^a^	42 (25–59)
**Gender**	
Male	2 (20.0)
Female	8 (80.0)
**Constituent Group**	
Community	4 (40.0)
Healthcare workers	2 (20.0)
Patients	2 (20.0)
Treatment partners	2 (20.0)
**Education Level**	
Less than primary school	0 (0)
Primary school	3 (30.0)
Junior secondary school	2 (20.0)
Senior secondary school	3 (30.0)
Higher than senior secondary school	2 (20.0)
**Employment Status** ^a^	
Employed	7 (77.8)
Unemployed	2 (22.2)

Note.

a Data missing for one participant

**Table 3:** Post intercare pilot survey responses by CFIR domain and construct

Item	Healthcare workers (total = 19) Agree *N* (%)	Community Members (total = 41) Agree *N* (%)	Treatment partners (total = 20) Agree *N* (%)

**Innovation Design (Overall Perception)**			
InterCARE would be successful in improving treatment of HIV-positive individuals with hypertension in my clinic.	18 (94.7)		20 (100.0)
InterCARE would be easy to understand and use after receiving training.	18 (94.7)		
InterCARE would have a visible and substantial impact on the health status of HIV-positive individuals with hypertension in my clinic.	16 (84.2)		
HIV-positive individuals with hypertension in my clinic would really benefit from InterCARE.	17 (89.5)		
Given how you understand InterCARE, how important do you think each component is: Provider Training^a^	16 (84.2)		
Given how you understand InterCARE, how important do you think each component is:	17 (89.5)		
Electronic Health Record^a^			
Given how you understand InterCARE, how important do you think each component is:	18 (94.7)		
Treatment partner^a^			
Combining high blood pressure and HIV care in the same clinic visit will improve a patient’s blood pressure.		35 (85.4)	
This program will be successful in improving patient knowledge about managing their own high blood pressure.		40 (97.6)	
**Innovation Compatibility**			
InterCARE is compatible and consistent with the needs of HIV-positive individuals with hypertension in my clinic.	15 (78.9)		
A treatment partner for high blood pressure will help patients remember to go to appointments.		40 (97.6)	20 (100.0)
A treatment partner for high blood pressure will help patients remember to take their medications.		40 (97.6)	19 (95.0)
Treatment partners will be successful in helping patients make changes to their diet to reduce blood pressure.		40 (97.6)	20 (100.0)
Treatment partners will be successful in helping patients increase their physical activity to reduce blood pressure.		40 (97.6)	19 (95.0)
A treatment partner will be able to teach patients about high blood pressure and how to manage their condition.			20 (100.0)
**Innovation Relative Advantage**			
InterCARE would be more effective than interventions we are currently using to manage hypertension in PLWH in my clinic.	18 (94.7)		
**Innovation Adaptability**			
It would be difficult to adapt InterCARE to meet the needs of different populations and groups of HIV-positive individuals with hypertension in my clinic.	9 (47.4)		
**Innovation Complexity**			
InterCARE is too complex to put into place in my clinic.	11 (57.9)		
Including treatment partners to help patients manage both HIV and high blood pressure would be too complicated.		15 (36.6)	10 (50.0)
Combining high blood pressure and HIV care into the same clinic visit would be too complicated.		29 (70.7)	
**Inner Setting Structural Characteristics**			
InterCARE would be problematic because we do not have enough HIV medical and supportive care resources to care for any additional HIV-positive patients with hypertension.	8 (42.1)		
InterCARE requires too many staff or other resources.	7 (36.8)		

**Table 4:** Interview themes by CFIR domain

CFIR Domain	Supporting Quotes for InterCARE project

Innovation	“I believe that this is an intervention that will benefit us as a society. It will benefit us because sometimes with these diseases a person can better take care of themselves when they have been educated and sensitized in their journey with living with their ailment. Yes, maybe just as you’ve said about having a supportive partner, I take it that a person must have someone beside them to help them with taking their medication when you were not able to go get them and also help remind you with your checkup days. So, I take it that this arrangement will be of much benefit to us.” (**Community member**) “In the recent time people have been receiving resources separately so I believe that just as they will start being provided all at the same time, it will help the health care workers provide services at a faster rate. In as much as these are different diseases, it is of utmost importance to consider observing them all at the same time. I believe that this will help doctors to help patients with faster ways that are more effective.” (**Community member**)
Outer Setting	“I have experienced discrimination as an HIV-positive person from people ever since I received treatment. This has made me not to disclose to anyone about my HIV status and that I am taking HIV treatment. Even when I was sick I did not tell anyone about my status and I was with my child who was my caretaker. I had noticed that my sister liked making fun of people with HIV/AIDS and she would go on about making fun of them whenever she gets drunk so that is why I wouldn’t share my status.” (**Treatment partner**) “The challenges, like I said; we are living in the rural areas, we have to travel. The problem will be now coming and paying for transport for 2 people that will be the challenge. They are engaged, this people are farmers, they move. The treatment partner will have to come at the same time with the client, so which means the social life is disturbed back at home.” (**Healthcare worker**) “I do not believe that there is anyone being discriminated against through this issue [hypertension]. In short, no I have never been to any place where I have seen anyone being discriminated against after discovering they have it [hypertension].” (**Community leader**)
Inner Setting	“Currently management of people living with HIV and Hypertension are in different departments so people with HIV are normally treated and managed at IDCC, and those with Hypertension are managed at outpatient department. So, the intervention I think will help bridge that gap so that this is possible is for them to be in one place.” (**Healthcare worker**) “Like you are saying the intervention will be continual to the ongoing implementation that is the use of electronics so the issue that may come is when there is lack of resources like internet, it would be difficult to continue to access patient information at a particular time when resources are limited and also the challenge is when there is lack of training to other staff. (**Healthcare worker**)
Individuals	“Yes, I think they will accept it. Because I believe doing things together is more efficient, when we do initiatives together, we go far.” (**Healthcare worker**) “The outcomes which will be important for staff, as we are doing this study, we are gaining knowledge. Some of which we did not have before, which I think will be a benefit to us in hypertension and co vascular diseases. For the section leaders I don’t know you mean the matron or the lady working at the IDCC, but it is the same, they are gaining skills and knowledge. We get to know more about management of hypertension and persons living with HIV/AIDS. I think for patients also, because now the program is not extended. They are taught more.” (**Healthcare worker**)
Implementation Process	“I take it that the most important thing in the village is the education, people need to be educated and be enlightened so that it is easier for them to be receptive of the interventions and initiatives. I take it that if there is the need for people to have a gathering at a Kgotla or even at the hospital it can happen so that people can be educated. Education is the only thing that can help people be receptive to such resources.” (**Community member**) “To make this intervention successful, its important to involve influential stakeholders, especially program leaders like the coordinators of various programs within the IDCC and the DHMT. Their active participation and support would significantly boost the effectiveness of this initiative.” (**Healthcare worker**)

## Data Availability

No datasets were generated or analysed during the current study.
