# Supplementary material for: Integrated Hypertension-HIV Care in Botswana: Mixed-Methods Evaluation of Determinants of Success in an Implementation Trial
Source: Glob Implement Res Appl. Author manuscript; Available in PMC 2026 Feb 27. (PMC12945319; doi:10.1007/s43477-025-00178-2)
Supplement: Supplemental material [file NIHMS2139079-supplement-Supplemental_material.docx]

**Supplementary Material**

***Supplemental File 1. Consolidated Criteria for Reporting Qualitative Research***

| **Topic** | **Guide Questions/Description** | | **Reported on**  **Page No.** | **Description** |
| --- | --- | --- | --- | --- |
| **DOMAIN 1: Research Team and Reflexivity** |  | |  |  |
| *Personal characteristics* |  | |  |  |
| 1. Interviewer/facilitator | Which author/s conducted the interview or focus groups? | | 10, lines 226-229 | “KBM (female, BSc in Health Education and Promotion), KM (female, Certificate in social work), TS (female, Bachelor of Public Health), and BN (male, Diploma in Health Education and Promotion).” |
| 1. Credentials | What were the researcher’s credentials | | 10, lines 226-229 | “KBM (female, BSc in Health Education and Promotion), KM (female, Certificate in social work), TS (female, Bachelor of Public Health), and BN (male, Diploma in Health Education and Promotion).” |
| 1. Occupation | What was their occupation at the time of the study | | 10, lines 226-229 | “All interviews were conducted in-person in a private room in the study clinic by **research assistants** …” |
| 1. Gender | Was the researcher male or female? | | 10, lines 226-229 | “KBM (female), KM (female,), TS (female), and BN (male).” |
| 1. Experience and training | What experience or training did the researcher have? | | 10, line 225-226 | “All interviews were conducted in-person in a private room in the study clinic by research assistants **trained in qualitative research with relevant expertise…”** |
| *Relationship with participants* |  | |  |  |
| 1. Relationship | Was a relationship established prior to study commencement. | | 10, line 229-230 | “The interviewers did not have prior relationships with the participants.” |
| 1. Participant knowledge of the interviewer | What did the participants know about the researcher? E.g., personal goals, reasons for doing the research | | 10, lines 218-219 | The intentions of the investigators were shared during this recruitment process. |
| 1. Interviewer characteristics | What characteristics were reported about the interviewer /facilitator | | 10, lines 226-229 | Nationality, gender, and credentials |
| Domain 2: Study Design |  | |  |  |
| *Theoretical framework* |  | |  |  |
| 1. Methodological orientation and theory | What methodological orientation was stated to underpin the study | |  | The Consolidated Framework for Implementation Research (CFIR) |
| *Participant selection* |  | |  |  |
| 1. Sampling | How were participants selected? E.g., purposive, convenience | | 8, line 183-184 | “These participants were purposively recruited based on their role in the community” |
| 1. Method of approach | How were participants approached? E.g., face-to-face, telephone, mail, email | | 9, lines 204  10, lines 225 | “Surveys were administered in-person…”  “All interviews were conducted in-person…” |
| 1. Sample size | How many participants were in the study? | | 12, line 270-273  12, lines 276 | Surveys  “One hundred participants completed the endline surveys. (Table 1).”  Interviews  “Ten participants completed interviews.” |
| 1. Non-participation | How many people refused to participate or dropped out? Reasons? | | 10, lines 219 | “Participant refusal and dropout did not occur.” |
| *Setting* |  | |  |  |
| 1. Setting of data collection | Where was the data collection? E.g., home, clinic, workplace | | 10, lines 225  9, lines 204 | “All interviews were conducted in-person in a private room in the **study clinic**”  “Surveys were administered in-person in a private room in the study clinics |
| 1. Prescence of non-participants | Was anyone else present besides the participants and researchers? | | 10, lines 225 | All interviews were conducted in-person **in a private room** in the study clinic |
| 1. Description of sample | What are the important characteristics of the sample? E.g., demographic data, date | | 31, 31 | Table 1 & table 2 |
| *Data collection* |  | |  |  |
| 1. Interview guide | Were questions, prompts, guides provided by the authors? Was it pilot tested? | |  | “Supplemental file 4” |
| 1. Repeat interviews | Were repeat interviews carried out? If yes, how many? | | N/A | No repeat interviews were carried out. |
| 1. Audio/visual recording | Did the research use audio or visual recording to collect the data? | | 10, line 232 | “All interviews were audio-recorded…” |
| 1. Field notes | Were field notes made during and/or after the interview of focus group? | | N/A | We have not reported on this. |
| 1. Duration | What was the duration of the interviews or focus group? | | 9, line 208 | “On average the interviews were 30-45 minutes long.” |
| 1. Data saturation | Was data saturation discussed? | | N/A | We have not reported on this. |
| 1. Transcripts returned | Were transcripts returned to participants of comment/or correction? | | N/A | “Transcripts were not returned to participants for comment or correction.” |
| **Domain 3: analysis and findings** | |  | | |
| Data analysis | |  | | |
| 1. Number of data coders | How many data coders coded the data? | | 12, Lines 264-265 | “…three members of the research team (AVP, KK, LB) reviewed all coded transcripts…” |
| 1. Description of the coding tree | Did authors provide a description of the coding tree? | | 11, lines 256-257 | “A deductive analysis was conducted. The codebook comprised constructs from CFIR, barriers, and facilitators that were identified *a priori*.” |
| 1. Derivation of themes | Were themes identified in advance or derived from the data? | | 11, lines 256-257 | “A deductive analysis was conducted. The codebook comprised constructs from CFIR, barriers, and facilitators that were identified *a priori*.” |
| 1. Software | What software, if applicable, was used to manage the data? | | 10, lines 232-234 | “The transcripts were uploaded into Dedoose (v9.0.107; Dedoose, 2023) for data management and analysis.” |
| 1. Participant checking | Did participants provide feedback on the findings? | | N/A | “Participants did not provide feedback on findings.” |
| *Reporting* |  | |  |  |
| 1. Quotations presented | Were participant quotations presented to illustrate the themes/findings? Was each quotation identified? E.g., participant number | | 34 | Table 4: Interview Themes by CFIR Domain |
| 1. Data and findings consistent | Was there consistence between the data presented and the findings? | |  | Yes. There is consistency between the data presented and the findings. |
| 1. Clarity of major themes | Were major themes clearly presented in the findings? | | 15-17 | Yes, major themes have been clearly presented in the findings. |
| 1. Clarity of minor theme | Is there a description of diverse cases of discussion of minor themes? | | 15-17 | Yes, there is a description of cases of minor themes. |

**Supplemental File 2**

***Standards for Reporting Implementation Studies (StaRI) Statement***

| **Checklist item** | **Page reported on** | **Implementation strategy** | **Page reported on** | **Intervention** |
| --- | --- | --- | --- | --- |
| 1. Title | 1 | Integrated hypertension in HIV care service in Botswana: a mixed-Methods Evaluation of Perspectives on Determinants of Success in a Pilot Type 2 Hybrid Effectiveness | | |
| 1. Abstract | 3 | InterCARE is evaluating the integration of hypertension treatment into HIV care in Botswana. This involves adapting the Electronic Health Records [EHR], health care workers training, and the use of treatment partners. A pilot hybrid type 2 effectiveness implementation trial in two public HIV clinics found high effectiveness, adoption, and fidelity overall but low adoption of EHR | | |
| 1. Introduction | 5 | Although evidence-based practices to manage hypertension exist in Botswana, studies report low awareness (46% aware of their hypertension diagnosis), low access to treatment (42% of diagnosed individuals on hypertension medication), and poor blood pressure control (44% of people with controlled blood pressure). Efforts to improve adoption of integrated care across the hypertension care cascade are needed in this setting. The InterCARE (Integrating Hypertension and Cardiovascular Care into Existing HIV Services Package) intervention was developed to increase the uptake of evidence-based practices for hypertension control among PWH in Botswana. | | |
| 1. Rationale | 6 | The Consolidated Framework for Implementation Research (CFIR) provides a framework for the systematic evaluation of factors that may affect implementation across multiple ecological levels. CFIR consists of five domains: innovation (e.g., components of InterCARE), inner setting (e.g., clinic leadership structure), outer setting (e.g., policy context, community stigma), individuals (e.g., self-efficacy), and implementation process (e.g., strategies). This research conducted contextual inquiry to provide insight into the success of InterCARE strategies and where adaptation was needed before scaling up InterCARE in a planned large-scale hybrid effectiveness implementation trial. | 6 | InterCARE integrates hypertension treatment into IDCCs through three evidenced-based strategies: 1) adaptation of the existing HIV EHR to capture hypertension risk and treatment, 2) facilitation of provider training on hypertension diagnosis and management, and 3) use of treatment partners to support adherence of hypertension care. Utilizing generalizable nomenclature from implementation science, the strategies align with the following strategies in the Expert Recommendations for Implementing Change (ERIC) compilation, a taxonomy of types of methods that can be used to enhance implementation of a clinical intervention: 1) “change record system,” 2) “conduct educational meetings,” and 3) “involve patients/consumers and family members” and “intervene with patients/consumers to enhance uptake and adherence, respectively.” |
| 1. Aims and objectives | 7 | This research conducted contextual inquiry to provide insight into the success of InterCARE strategies and where adaptation was needed before scaling up InterCARE in a planned large-scale hybrid effectiveness implementation trial. |  |  |
| 1. Design | 11 | Convergent mixed method |  |  |
| 1. Context | 7 | To optimize understanding of contextual factors, HIV clinics were stratified by size (e.g., small rural serving less than 500 patients versus large peri-urban serving more than 1,500 patients) and urbanicity. Two HIV clinics, one from each of the stratification categories, were randomly selected for participation in the study: S clinic (small clinic serving 500 patients in the rural south of Botswana) and L clinic (large clinic serving more than 3,000 patients in the peri-urban Northeast of Botswana). S clinic primarily employs nurses, whereas L clinic employs general nurses, physicians (medical officers), and nurse practitioners (including HIV nurse prescribers). |  |  |
| 1. Targeted ‘sites’ | 7 | Two HIV clinics, one from each of the stratification categories, were randomly selected for participation in the study: S clinic (small clinic serving <500 patients in the rural south of Botswana) and L clinic (large clinic serving more than >1,500 patients in the peri-urban Northeast of Botswana). S clinic primarily employs nurses, whereas L clinic employs general nurses, physicians (medical officers), and nurse practitioners (including HIV nurse prescribers). | 8 | To capture the diversity of perspectives, participants were recruited from four constituent groups: community members (e.g., local leaders, council members, and leaders in the commercial sector), healthcare workers, patients, and HIV treatment partners of the same patients. Participants were required to be at least 20 years old. Patients were defined as individuals with a dual diagnosis of both HIV and hypertension who were enrolled for care at one of the study sites. All eligible patients, treatment partners, and healthcare workers were consecutively recruited in-person at each site until the target sample was achieved. Community members were eligible if they attended one of the study sites but were not InterCARE participants or treatment partners. These participants were purposively recruited based on their role in the community until the target sample was reached at each study site. Sampling aimed to recruit more community members than other constituent groups, as their perspective was particularly important for adaptations to InterCARE in future scale-up. Participants who completed the endline surveys differed from participants who completed the baseline surveys. |
| 1. Description | 6 | InterCARE integrates hypertension treatment into HIV clinics through three evidenced-based strategies: 1) adaptation of the existing HIV EHR to capture hypertension risk and treatment, 2) facilitation of provider training on hypertension diagnosis and management, and 3) use of treatment partners to support adherence of hypertension care. |  |  |
| 1. Sub-groups | 8 | To capture the diversity of perspectives, participants were recruited from four constituent groups: community members (e.g., local leaders, council members, and leaders in the commercial sector), healthcare workers, patients (PLWH & hypertension), and HIV treatment partners for PLWH & hypertension (these are community based supporters, not necessarily in an intimate relationship) of the same patients. Participants were required to be at least 20-75 years old. Patients were defined as individuals with a dual diagnosis of both HIV and hypertension who were enrolled for care at one of the study sites. All eligible patients, treatment partners, and healthcare workers were consecutively recruited in-person at each site until the target sample was achieved. Community members were eligible if they attended one of the study sites but were not InterCARE participants or treatment partners. |  |  |
| 1. Outcomes |  | -Proportion of PLWH with diagnosed hypertension receiving anti-hypertensive medication with controlled blood pressure  -Proportion of clinic encounters in EHR where anti-hypertensive medications are prescribed if indicated |  |  |
| 1. Process Evaluation |  | N/A |  |  |
| 1. Economic Evaluation |  | N/A |  |  |
| 1. Sample size | 12 | Surveys  “One hundred participants completed the endline surveys. (Table 1).”  Interviews  “Ten participants completed interviews.” |  |  |
| 1. Analysis | 11 | A convergent mixed methods analysis approach was used. First, the survey and interview data were analyzed separately. Descriptive statistics were calculated on participant demographic data for both the surveys and interviews, as well as survey data on agreement stratified by constituent group. Fishers exact test was used to compare the baseline data to the endline data on surveys. For interviews a deductive analysis was conducted. The codebook comprised constructs from CFIR, barriers, and facilitators that were identified *a priori*. An initial coding scheme was applied to two transcripts by two independent coders. statistical software R (v4.3.2; R Core Team, 2023).  Given the small sample size, double coding of 20% of the transcripts included the two initial transcripts to ensure consistency and refine the coding framework before proceeding with the full analysis. Disagreements were resolved through team discussions and after resolving them the pair achieved reliability (Cohen’s Kappa = 0.86). The final coding scheme was applied to all transcripts by one investigator. However, three members of the research team reviewed all coded transcripts to confirm the accuracy of themes. All constituent groups were analyzed together using Dedoose (v9.0.107; Dedoose, 2023) for data management and analysis. | | |

**Supplemental table 3**

***Overview of participant groups, recruitment methods, and relationship to baseline and 12-month follow-up timepoints***

| **Participant Group** | **Data type** | **Recruitment timepoint** | **Recruitment method** |
| --- | --- | --- | --- |
| Patients (HIV & HTN) | Quantitative | Baseline & endline | Purposive sampling at HIV clinics |
| Treatment Partners | Quantitative | Baseline & endline | Purposive sampling at HIV clinics |
| Healthcare Workers | Quantitative | Baseline & endline | Recruited from study sites |
| Community Members (Community Leaders & business community) | Quantitative | Baseline & endline | Purposively selected based on community role |
|  |  |  |  |
| Patients (for Interviews) | Qualitative | Baseline & endline | Purposive sampling from 12-month retained cohort |
| Treatment Partners (Interviews) | Qualitative | Baseline & endline | Purposively selected, retained at 12 months |
| Healthcare Workers (Interviews) | Qualitative | Baseline & endline | Purposively selected from providers involved in InterCARE |
| Community Members (Community Leaders & business community) | Qualitative | Baseline & endline | Purposively selected based on community role |

**Supplemental File 4**

***Sample Survey Instrument***

Integrating Hypertension and Cardiovascular Diseases Care into The Existing HIV Services Package In Botswana. (InterCARE) PRE-IMPLEMENTATION SURVEY (Treatment Partner-Mopati)

Goal of the survey:

- Understand the experience of treatment partners related to HIV and HTN
- Understand what they view as areas for improvement both related to existing programs and the InterCARE intervention
- Explore their attitudes towards integrating HIV and HTN care

**SECTION 1: Baseline Information**

1. Age……………….
2. Gender: a. Female b) Male c) Other d) Refuse to answer
3. Highest level of school completed
   1. Less than Primary (Including Non formal education)
   2. Primary School (Standard 1-7)
   3. Junior Secondary (Form 1-3)
   4. Senior Secondary (Form 4-5)
   5. Higher than senior secondary (university, diploma, etc.)
4. Are you currently working?
   1. Yes [jump to question 5]
   2. No [jump to question 6]
5. In your main job what type of work do you do?
   1. Occasional or Casual employment (piece job)
   2. Seasonal employment
   3. Formal wage employment (full-time)
   4. Formal wage employment (part-time)
   5. Self-employed in agriculture
   6. Self-employed making money, full time
   7. Self-employed making money, part time
   8. Other
6. What is the reason why you are not working?
   1. Waiting to continue agricultural work
   2. Unemployed (looking for work)
   3. Unemployed (waiting to start new work
   4. Unable to work (permanently sick or injured)
   5. Student/ Apprentice/ Volunteer
   6. Housewife/ Homemaker (not looking for work)
   7. Retired
   8. Other
   9. Not looking for work
7. Monthly Household Income
8. < 1000
9. 1001 - 5000
10. 5001 - 10,000
11. 10,001 & above
12. Prefer not to answer
13. I don’t know
14. Number of members (including participant) living in the household? __________
15. Does any member of this household have any of the following that are currently working? (check all that apply).
    - Radio
    - Don't want to answer
    - TV
    - Landline telephone
    - Cell phone
    - Computer
    - Access to internet
    - Refrigerator
16. Does any member of this household (excluding visitors) own any of the following forms of transport in working condition? (check all that apply).

- Motor vehicle (car,truck,taxi, etc)
- Tractor
- Bicycle
- Motorcycle/scooter
- Donkey or cow cart
- Donkey/horses

1. What is the main source of energy used for cooking?

- Charcoal/wood
- Paraffin
- Gas
- Electricity (mains)
- Electricity (solar)
- No cooking done
- Other

1. How many years have you been a treatment partner for HIV?
   1. < 1 year
   2. 1-2 years
   3. 3-5 years
   4. > 5 years
2. Do you live in the same household as your peer with HIV and high blood pressure?
   1. Yes
   2. No
3. What is your relationship to your peer with HIV?
   1. Spouse (Husband or Wife)
   2. Child (Son or Daughter)
   3. Parent (Mother or Father)
   4. Sibling (Brother or Sister)
   5. Other Blood Relative (Aunt, Uncle, Grandparent, Cousin)
   6. Friend/Acquaintance
   7. Other __________________

**Hypertension Literacy Questionnaire**

1. If someone’s blood pressure is 160/90 is it:
   1. High
   2. Normal
   3. Low
   4. I don’t know
2. Decreasing which of the following will be most helpful in reducing blood pressure:
   1. Fizzy drinks
   2. Spinach (Morogo)
   3. Salt
   4. Sugar
   5. I don’t know
3. Treatment for high blood pressure can reduce the risk for which of the following?
   1. Pneumonia
   2. Stroke
   3. Diabetes
   4. Liver disease
   5. I don’t know
4. Which of the following can help reduce blood pressure?
   1. Doing less exercise
   2. Losing weight
   3. Drinking more milk
   4. Checking the blood pressure every day
   5. I don’t know
5. For how long do most people need to take blood pressure medication?
   1. Until the high blood pressure is cured
   2. Until they reach the age of 90 years
   3. Life-long
   4. I Don’t know

**SECTION 2: HIV Care: LEAD IN QUESTIONS**

*****Order questions**

|  | Strongly Agree | Agree | Neither Agree or Disagree | Disagree | Strongly Disagree | No Answer |
| --- | --- | --- | --- | --- | --- | --- |
| I have received adequate training as a HIV treatment partner |  |  |  |  |  |  |
| I am confident I have the knowledge and skills required to be at treatment partner for HIV |  |  |  |  |  |  |
| I need additional support to complete the job I am expected to do as a treatment partner for HIV |  |  |  |  |  |  |
| I am expected to do too many things as a treatment partner for HIV |  |  |  |  |  |  |

1. For the following section, read the statement and inform us if you: Strongly agree, Agree, Neither Agree or Disagree, Disagree, strongly disagree

**SECTION 3: High Blood Pressure (HTN) Care**

For the following section, think about your role as a treatment partner. As a treatment partner for high blood pressure, read the statement and inform us if you: Strongly agree, Agree, Neither Agree or Disagree, Disagree, Strongly Disagree

Attitudes for Adoption and selected CFIR: ARTAS question (modified)

|  | ***CFIR construct*** | ***Strongly Disagree*** | ***Disagree*** | ***Neither Agree nor Disagree*** | ***Agree*** | ***Strongly Agree*** | ***No Answer*** |
| --- | --- | --- | --- | --- | --- | --- | --- |
| 1. *Combining high blood pressure and HIV care in the same clinic visit will improve the blood pressure of my peer* | *Intervention* |  |  |  |  |  |  |
| 1. *Including treatment partners to help patients manage both HIV and high blood pressure would be too complicated* | *Intervention* |  |  |  |  |  |  |
| *A treatment partner for high blood pressure will help patients remember to go to appointments.* | *Intervention* |  |  |  |  |  |  |
| 1. *A treatment partner for high blood pressure will help patients remember to take their medications.* | *Intervention* |  |  |  |  |  |  |
| 1. *A treatment partner will be able to teach patients about high blood pressure and how to manage their condition* | *Intervention* |  |  |  |  |  |  |
| 1. *Treatment partners will be successful in helping patients make changes to their diet to reduce blood pressure* | *Intervention* |  |  |  |  |  |  |
| *Treatment partners will be successful in helping patients increase their physical activity to reduce blood pressure* | *Intervention* |  |  |  |  |  |  |

Score *Note. 1 = Strongly Disagree, 2 = Disagree, 3 = Neither Agree nor Disagree, 4 = Agree, 5 = Strongly Agree*.

*Items were reverse scored prior to conducting t-tests and creation of average score

**Supplemental File 5. Sample Semi-Structured Interview Guide**

## Integrating Hypertension and Cardiovascular Diseases Care into The Existing HIV Services Package In Botswana. (InterCARE)

## INTERVIEW QUESTIONS (Patient)

***SECTION ONE***

Do I have your permission to interview you regarding your thoughts on high blood pressure management and the intervention we are planning?

Do I have your permission to record this interview? *(If YES, turn on digital recorder and state participant ID before starting the interview)*

***PARTICIPANT BACKGROUND INFORMATION***

***First, we would like to know a little about you.***

1. Age……………….
2. Gender: a. Female b) Male c) Other d) Refuse to answer
3. Highest level of school completed
   1. Less than Primary (Including Non formal)
   2. Primary School (Standard 1-7)
   3. Junior Secondary (Form 1-3)
   4. Senior Secondary (Form 4-5)
   5. Higher than senior secondary (university, diploma, etc.)
4. What is the main source of energy used for cooking?
5. Charcoal/wood
6. Paraffin
7. Gas
8. Electricity (mains)
9. Electricity (solar)
10. No cooking done
11. Other
12. Does any member of this household have any of the following that are currently working? (check all that apply).

- Radio
- Don't want to answer
- TV
- Landline telephone
- Cell phone
- Computer
- Access to internet
- Refrigerator

6. Does any member of this household (excluding visitors) own any of the following forms of transport in working condition? (check all that apply).

- Motor vehicle (car, truck, taxi, etc)
- Tractor
- Bicycle
- Motorcycle/scooter
- Donkey or cow cart
- Donkey/horses

**PATIENT BACKGROUND INFORMATION**

**We will now ask you some questions about the services you have received at this clinic in the past.**

1. Where do you go most frequently to get HIV care?
2. What have your experiences been working with a treatment partner (Mopati) for your HIV care?
   1. What are the benefits?
   2. What are the challenges?
3. What is your relationship to your treatment partner (e.g Is he or she a family member or close friend/acquaintance/household member)?
   1. What are some of the benefits of having this type of relationship/connection with your treatment partner?
   2. What are some of the challenges?
4. What have your experiences been getting counseling at the clinic for your HIV?
   1. What have been the benefits of HIV counseling?
   2. What improvements can be made regarding HIV counseling?
5. Where do you go most frequently to get care for your high blood pressure? Is it the same location as where you get HIV care? ***SECTION TWO:***

***INNER SETTING***

***We will now ask you some questions regarding your diagnosis of HTN and experiences having HTN.***

***Need for Change***

1. When were you diagnosed with high blood pressure and how was the diagnosis made?
   1. *Probe: [What was your experience? How was the diagnosis communicated to you? How easily did you understand this diagnosis?]*
2. What are some things that have made it easier and helped you to manage your high blood pressure?
3. What are some of the challenges you face in managing your high blood pressure?
4. How has it been taking blood pressure medications? If not taking medications, can skip?
   1. What has made it easy or hard to get your medications
5. Who gives you information and support in the clinic to manage your blood pressure?
   1. Probe: Do you spend enough time with this person (nurse or doctor)?
   2. How would you describe your interactions? Have they been beneficial? Have they helped you follow the recommendations for managing high blood pressure?
6. Who is a person at home or in your community that you rely on to help you manage your blood pressure [can be more than one person]? Probe: Is he or she your Mopati for HIV?
   1. What aspects of your high blood pressure care does this person help you with? What are the benefits of having a person to help you manage your blood pressure?
   2. Are there aspects of your high blood pressure care this person is unable to help you with?
7. What have your experiences been getting counseling for your high blood pressure at this clinic in the past 12 months? *[Prompt: This counseling may be related to lifestyle changes to manage your high blood pressure (diet modifications, increasing physical activity) or if you are on medications - taking your medications regularly and monitoring for side effects]*
   1. What have been benefits of this counseling? What improvements can be made regarding high blood pressure management counseling?
8. How do you get care for both HIV and high blood pressure?
   1. *Probe: Is this done at the same time in a single clinic visit? In a single day? By the same provider?*
   2. How is the care you get for HIV and high blood pressure similar or different?
      1. *[Probe: Is getting medications for HIV and high blood pressure similar or different?]*
      2. *[Probe: Is the way you get counseling similar or different?]*
9. *[For this question, you want to probe the patient based on their responses to the question above. Example: If they responded that they receive HIV and HTN care at different clinic locations, different clinic visits, or by different providers, you can ask if they would prefer receiving care for both diseases in the same visit].* We would like to know more about how you receive care for both of your HIV and high blood pressure.
   1. What works well about how you are receiving HIV care? High blood pressure care? How well coordinated are they (by coordination we mean receiving HIV and high blood pressure care in the same clinic visit by the same provider; also, receiving medications for both medical conditions at the same pharmacy)
   2. *[Probe: Do you prefer that they are separated with different providers? Or, would you prefer to see one provider who manages and counsels you on both HIV and high blood pressure in the same clinic visit?]*

INTERVENTION CHARACTERISTICS

Next, we are interested in finding out your views of the InterCare intervention presented. Specifically, whether you think it might be an improvement in how you are currently getting care and support to help you manage your HIV and high blood pressure; to remind you, InterCare is a program that will integrate care for high blood pressure with HIV care. You will see a single provider in the same clinic visit to receive care for both HIV and high blood pressure. The major components of this intervention include: 1) Training and education for health care providers on hypertension and cardiovascular disease. 2) an electronic medical record that will help health care providers keep track of the blood tests you require and when you need refills on your medications. 3) Using a Mopati to help you manage high blood pressure and HIV.

1. What is your opinion about the program we are proposing overall? What would make things better? What do you the challenges might be?
   1. *[Probe: What might the benefits be to you? The barriers? What should the goals of this program be?]*
   2. *[Probe: Do you think this program will improve access to services? Improve access to doctors and nurses? Reduce wait times? Help reduce travel time and expense? Improve patient education and counseling? ]*
2. What is your opinion specifically about combining high blood pressure and HIV care into a single clinic visit?
   1. *[Probe-How would this be a change from how you are currently receiving care? Would it be better if it is? If not a change, what are other things that would be helpful in improving your high blood pressure and HIV care?]*
   2. What are some of the barriers to combining high blood pressure and HIV care into a single clinic visit?
3. We will now ask a couple questions about your opinion on peers to support high blood pressure management.
   1. What is your opinion about having a peer to help you with high blood pressure management? What might the benefits be? The barriers? What do you think the job of this peer should be? What aspects of high blood pressure care could they help with?

***Relative Advantage***

1. We have described the program we are planning on starting in this clinic. What else could make it easier for you to receive care for and manage your HIV and your blood pressure?

***Knowledge and Beliefs - Individual***

1. Have you encountered stigma associated with you having high blood pressure? By stigma I mean discrimination against you or disapproval of you by other people because you have high blood pressure.
   1. If yes, what can be done to decrease this stigma in the community? At the clinic?

***SECTION FOUR***

***PROCESS***

**Through these final questions, we would like to find out how this intervention will affect work processes in your facility. We are also interested in your opinion of whose buy-in, input and expertise is needed to implement and maintain the intervention.**

***Key Stakeholders***

1. How should information about this intervention be communicated with you as a patient? (e.g e-mails, brochures, public announcements at churches, schools, kgotla?)
   1. Who should communicate this information to you (e.g. local leaders/chiefs, doctors, nurse)?
2. Is there another program or idea you have that you think would be better than the program we are describing?
   1. [If Yes] Can you describe this program or idea?
   2. [If Yes] Why would people prefer the alternative program or idea?
3. We are about to finish the interview. Is there anything else you would like to add about the proposed program to improve both HIV and high blood pressure care in this clinic?

**End of the Interview. Thank you for participating in this interview.**

**Supplemental File 6**

***Pre-Post Comparison of Survey Responses by CFIR Construct***

| **Table 5. Pre-Post Comparison of Survey Responses by CFIR Construct** | | | | | | | | | |
| --- | --- | --- | --- | --- | --- | --- | --- | --- | --- |
|  | **HCWs**  Agree N (%) | | | **Community**  Agree N (%) | | | **Treatment partners**  Agree N (%) | | |
| **Item** | **Pre** | **Post** | **p-value** | **Pre** | **Post** | **p-value** | **Pre** | **Post** | **p-value** |
| **Innovation Design (Overall Perception)** | | | | | | | | | |
| InterCARE would be successful in improving treatment of HIV-positive individuals with HTN in my clinic. | 18(90.0) | 18  (94.7) | >0.99 |  |  |  | 16(35.0) | 20(100.0) | 0.11 |
| InterCARE would be easy to understand and use after receiving training. | 17(85.0) | 18  (94.7) | 0.61 |  |  |  |  |  |  |
| InterCARE would have a visible and substantial impact on the health status of HIV-positive individuals with HTN in my clinic. | 18(90.0) | 16  (84.2) | 0.66 |  |  |  |  |  |  |
| HIV-positive individuals with HTN in my clinic would really benefit from InterCARE. | 19(95.0) | 17  (89.5) | 0.61 |  |  |  |  |  |  |
| Given how you understand InterCARE, how important do you think each component is: Provider Training^a^ | 15(75.0) | 16  (84.2) | 0.69 |  |  |  |  |  |  |
| Given how you understand InterCARE, how important do you think each component is: Electronic Health Record^a^ | 13(65.0) | 17  (89.5) | 0.13 |  |  |  |  |  |  |
| Given how you understand InterCARE, how important do you think each component is: Treatment partner^a^ | 16(80.0) | 18  (94.7) | 0.34 |  |  |  |  |  |  |
| Combining high blood pressure and HIV care in the same clinic visit will improve a patient’s blood pressure. |  |  |  | 38  (95.0) | 35  (85.4) | 0.26 |  |  |  |
| This program will be successful in improving patient knowledge about managing their own high blood pressure. |  |  |  | 39  (97.5) | 40  (97.6) | >0.99 |  |  |  |
| **Innovation Compatibility** | | | | | | | | | |
| InterCARE is compatible and consistent with the needs of HIV-positive individuals with HTN in my clinic. | 18(90.0) | 15  (78.9) | 0.41 |  |  |  |  |  |  |
| A treatment partner for high blood pressure will help patients remember to go to appointments. |  |  |  | 38  (95.0) | 40  (97.6) | 0.62 | 19(95.0) | 20  (100.0) | >0.99 |
| A treatment partner for high blood pressure will help patients remember to take their medications. |  |  |  | 39  (97.5) | 40  (97.6) | >0.99 | 20  (100.0) | 19(95.0) | >0.99 |
| Treatment partners will be successful in helping patients make changes to their diet to reduce blood pressure. |  |  |  | 38  (95.0) | 40  (97.6) | 0.62 | 20  (100.0) | 20  (100.0) | >0.99 |
| Treatment partners will be successful in helping patients increase their physical activity to reduce blood pressure. |  |  |  | 39  (97.5) | 40  (97.6) | >0.99 | 19(95.0) | 19(95.0) | >0.99 |
| A treatment partner will be able to teach patients about high blood pressure and how to manage their condition. |  |  |  |  |  |  | 19(95.0) | 20  (100.0) | >0.99 |
| **Innovation Relative Advantage** | | | | | | | | | |
| InterCARE would be more effective than interventions we are currently using to manage HTN in PLWH in my clinic. | 17(85.0) | 18  (94.7) | 0.61 |  |  |  |  |  |  |
| **Innovation Adaptability** | | | | | | | | | |
| It would be difficult to adapt InterCARE to meet the needs of different populations and groups of HIV-positive individuals with HTN in my clinic. | 12(60.0) | 9(47.4) | 0.53 |  |  |  |  |  |  |
| **Innovation Complexity** | | | | | | | | | |
| InterCARE is too complex to put into place in my clinic. | 13(65.0) | 11  (57.9) | 0.75 |  |  |  |  |  |  |
| Including treatment partners to help patients manage both HIV and high blood pressure would be too complicated. |  |  |  | 25  (62.5) | 15  (36.6) | **0.03** | 8(40.0) | 10(50.0) | 0.75 |
| Combining high blood pressure and HIV care into the same clinic visit would be too complicated. |  |  |  | 27  (67.5) | 29  (70.7) | 0.81 |  |  |  |
| **Inner Setting Structural Characteristics** | | | | | | | | | |
| InterCARE would be problematic because we do not have enough HIV medical and supportive care resources to care for any additional HIV-positive patients with HTN. | 11(55.0) | 8(42.1) | 0.53 |  |  |  |  |  |  |
| InterCARE requires too many staff or other resources. | 6(30.0) | 7(36.8) | 0.74 |  |  |  |  |  |  |
